# UPFront and center in RNA decay: UPF1 in nonsense-mediated mRNA decay and beyond

**DOI:** 10.1261/rna.070136.118

**Published:** 2019-04

**Authors:** Yoon Ki Kim, Lynne E. Maquat

**Affiliations:** 1 Creative Research Initiatives Center for Molecular Biology of Translation, Korea University, Seoul 02841, Republic of Korea; 2 Division of Life Sciences, Korea University, Seoul 02841, Republic of Korea; 3 Department of Biochemistry and Biophysics, School of Medicine and Dentistry, University of Rochester, Rochester, New York 14642, USA; 4 Center for RNA Biology, University of Rochester, Rochester, New York 14642, USA

**Keywords:** nonsense-mediated mRNA decay, Staufen-mediated mRNA decay, UPF1

## Abstract

Nonsense-mediated mRNA decay (NMD), which is arguably the best-characterized translation-dependent regulatory pathway in mammals, selectively degrades mRNAs as a means of post-transcriptional gene control. Control can be for the purpose of ensuring the quality of gene expression. Alternatively, control can facilitate the adaptation of cells to changes in their environment. The key to NMD, no matter what its purpose, is the ATP-dependent RNA helicase upstream frameshift 1 (UPF1), without which NMD fails to occur. However, UPF1 does much more than regulate NMD. As examples, UPF1 is engaged in functionally diverse mRNA decay pathways mediated by a variety of RNA-binding proteins that include staufen, stem–loop-binding protein, glucocorticoid receptor, and regnase 1. Moreover, UPF1 promotes tudor-staphylococcal/micrococcal-like nuclease-mediated microRNA decay. In this review, we first focus on how the NMD machinery recognizes an NMD target and triggers mRNA degradation. Next, we compare and contrast the mechanisms by which UPF1 functions in the decay of other mRNAs and also in microRNA decay. UPF1, as a protein polymath, engenders cells with the ability to shape their transcriptome in response to diverse biological and physiological needs.

## INTRODUCTION

The ATP-dependent RNA helicase upstream frameshift 1 (UPF1) is required for the nonsense-mediated mRNA decay (NMD) of all eukaryotic NMD targets studied to date (Brogna et al. 2016; Hug et al. 2016; Karousis et al. 2016; Saveanu and Jacquier 2016; Celik et al. 2017; Karousis and Mühlemann 2018; Raimondeau et al. 2018). It has been known for almost 20 yr that the abundance of UPF1 in human HeLa cells is ∼10-fold higher than the abundance of the two other UPF proteins—UPF2 and UPF3X (also called UPF3B)—that function in NMD (Maquat and Serin 2001), offering an early indication that UPF1 functions may extend beyond NMD. Since that realization, ample evidence has made it clear that UPF1 multitasks by contributing to other RNA decay pathways. Additionally, the finding that UPF1 can function as an E3-ubiquitin ligase that represses myogenesis provides an example of how UPF1 function in NMD can be coordinated with its function in protein decay (Takahashi et al. 2008; Feng et al. 2017)—a link that will undoubtedly be extended in the future to other cellular processes.

To date, UPF1-dependent RNA decay pathways include NMD, staufen (STAU)-mediated mRNA decay (SMD), replication-dependent histone mRNA decay (HMD), glucocorticoid receptor-mediated mRNA decay (GMD), regnase 1-mediated mRNA decay (RMD), and tudor-staphylococcal/micrococcal-like nuclease (TSN)-mediated microRNA decay (TumiD). Future studies will undoubtedly discover additional pathways. Given that UPF1 binds to all physically accessible transcripts in cells (Hogg and Goff 2010; Hurt et al. 2013; Zünd et al. 2013; Kurosaki et al. 2014), the participation of UPF1 in specific RNA decay pathways means that UPF1 must be purposefully recruited to and thereby activated on each type of substrate. In this review, we outline how substrates of each UPF1-dependent RNA decay pathway recruit and utilize UPF1 for their very distinct purposes.

## NMD: DUAL ROLES OF UPF1 IN CONTROLLING THE QUALITY OR QUANTITY OF GENE EXPRESSION

It is important that eukaryotic genes be expressed at the right time and place, and at an appropriate level. However, gene expression is not without errors: Abnormally synthesized gene products routinely arise as a consequence of mistakes made during gene replication, gene transcription, pre-mRNA processing, and/or mRNA translation. These mistakes can result in the production of improperly functional or nonfunctional proteins that could be deleterious to cellular metabolism. Thus, aberrant gene products would ideally be detected and eliminated by cells as a means to increase the fidelity of gene expression, thereby ensuring homeostasis. To this end, eukaryotic cells have evolved highly sophisticated mechanisms of quality control.

An estimated 5%–30% of human transcripts are faulty because they harbor a premature termination codon (PTC; Bhuvanagiri et al. 2010; Huang and Wilkinson 2012; Nguyen et al. 2014), some of which may yield truncated polypeptides. Fortunately, all eukaryotic cells that have been examined have developed nonsense-mediated mRNA decay (NMD) for quality control. NMD recognizes and eliminates PTC-containing mRNAs as a means to reduce the production of aberrant and potentially toxic proteins. NMD also eliminates the PTC-containing mRNAs that typify an estimated 30% of genetic or acquired diseases in humans. For these affected individuals, disease is due to the absence of full-length functional protein and, if genetic, is recessively inherited. However, many people with dominantly inherited diseases harbor a PTC that fails to trigger NMD. In this case, disease is due to the production of a truncated protein that is detrimental to cell function even when the other allele is normal and expressed (Bhuvanagiri et al. 2010; Huang and Wilkinson 2012; Nguyen et al. 2014).

In addition to its quality-control role, NMD also regulates the stability of ∼5%–10% of normal, physiologic mRNAs. This is exemplified by the many developmental and environmental changes that reduce the efficiency of NMD so that natural NMD targets are expressed. Among these NMD targets are groups of mRNAs producing proteins that promote the appropriate cellular response (Kurosaki et al. 2019). In many cases, the efficiency of NMD is reduced in response to environmental change by a mechanism that inhibits UPF1 function in the pathway. For example, an increased ratio of STAU1 relative to UPF2 results in more SMD and less NMD during myogenesis since each protein competes for binding to UPF1 (Gong et al. 2009). Among the group of NMD targets stabilized are mRNAs whose encoded proteins promote the maturation of myoblasts to multinucleated myotubes (Gong et al. 2009). As another example, microRNAs that target UPF1 mRNA inhibit UPF1 production during neurogenesis so as to promote differentiation from the stem-cell state to the neural state (Bruno et al. 2011; Lou et al. 2014). As a final example, severe DNA damage by chemotherapeutics decreases the efficiency of NMD because UPF1 is cleaved by caspases, thereby promoting apoptosis (Jia et al. 2015; Popp and Maquat 2015). The reader is referred to Kurosaki et al. (2019) for additional ways in which changes in the efficiency of NMD by targeting UPF1 function contribute to cellular adaptation. Most notably among them, NMD offers anti-viral mechanisms that viruses often counter by sequestering, inactivating, or down-regulating the abundance of UPF1. In addition, depending on how cancers evolve, UPF1 may be mutated (e.g., see Liu et al. 2014) or up-regulated (El-Bchiri et al. 2008; Bokhari et al. 2018) to promote tumor-cell survival.

## NMD: RECOGNITION OF NMD SUBSTRATES

**FIGURE 1.**
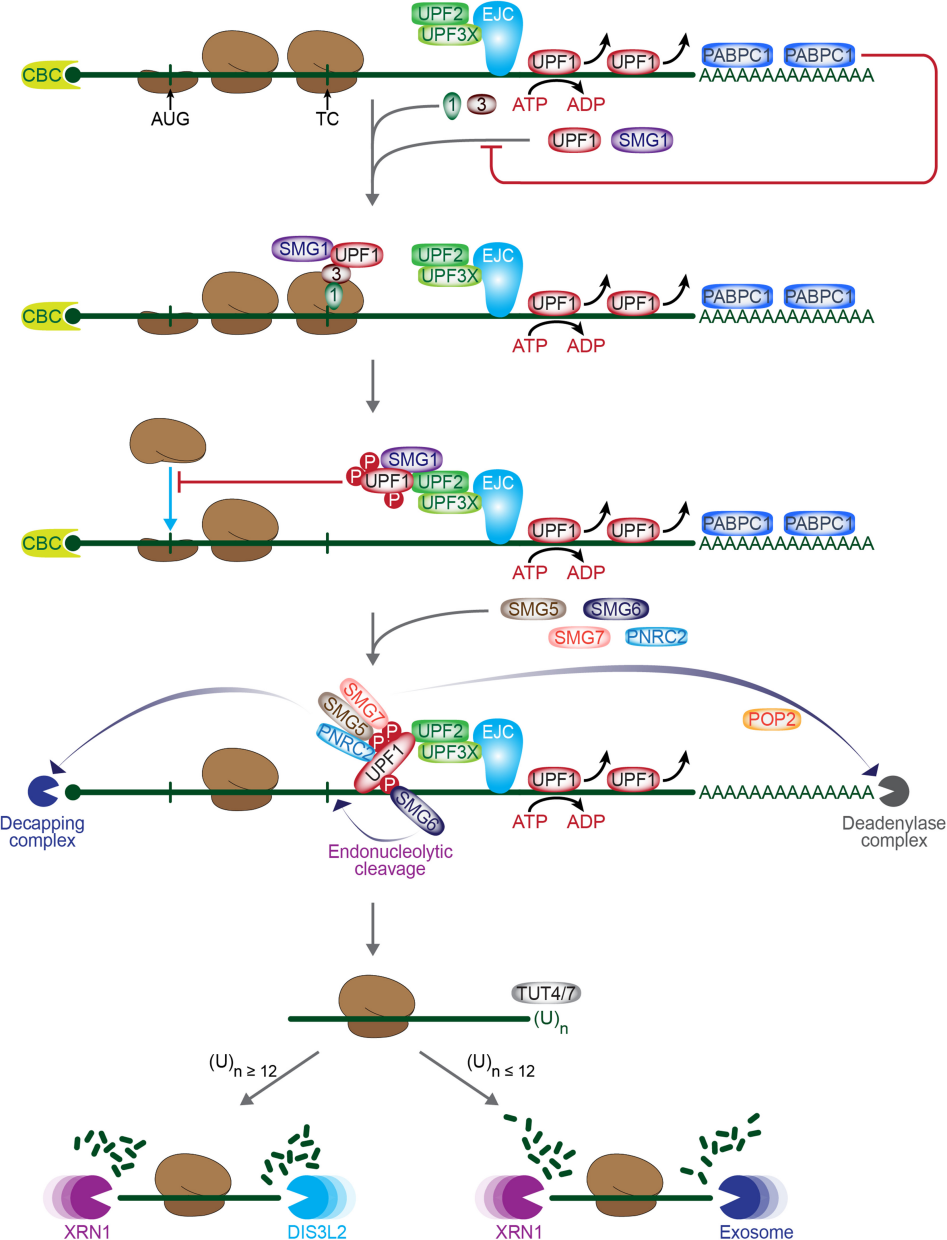
Stepwise processes for nonsense-mediated mRNA decay (NMD). Whether a termination codon (TC) does or does not trigger NMD is determined by two opposing events, respectively, termination-delaying (i.e., NMD-stimulating) events, which are promoted by factor(s) such as a 3′UTR EJC or a structured 3′UTR, each of which has a propensity for binding the UPF1 ATP-dependent RNA helicase, or termination-promoting (i.e., NMD-antagonizing) factor(s) such as PABPC1. Shown here are the steps that constitute 3′UTR EJC-dependent NMD. Briefly, when a 3′UTR EJC remains after translation termination at a TC, UPF1, its kinase SMG1 (and additional SMG factors that will not be discussed here) form the SURF complex together with the two termination factors eRF1 and eRF3. Subsequently, UPF1 and SMG1 either bridge or move to the EJC, at which point EJC-bound UPF2 binding to the CH domain of UPF1 induces a large conformational change in UPF1, concomitantly promoting the phosphorylation of UPF1 by the SMG1 kinase and, possibly, also promoting its helicase activity. Phosphorylated UPF1 represses translation by precluding further translation initiation events and also recruits factors that either directly (SMG6) or indirectly (SMG5−SMG7 and/or PNRC2) result in decay. Endoribonucleolytic cleavage occurs near the TC by SMG6, whereas DCP2−DCP1A decapping followed by 5′-to-3′ exoribonucleolytic activities are recruited by PNRC2, and CCR4−POP2 deadenylating as well as 3′-to-5′ exosome activities are recruited by SMG5−SMG7. Ribosome-bound NMD decay intermediates can be uridylated at their 3′-ends by TUT4 and TUT7 to promote further 3′-to-5′ exoribonucleolytic by DIS3L2 and/or the exosome. Terminal uridylation can also induce decapping followed by XRN1-mediated 5′-to-3′ degradation.

NMD serves two masters in cells: One promotes the quality of gene expression, and the other promotes adaptation to changing environments by regulating the quantity of gene expression. For each purpose, the NMD pathway consists of two essential steps: substrate recognition, and substrate degradation (Fig. 1). Despite extensive studies of each step, some details remain obscure. We overview a basic model for the two steps from the vantage point of UPF1, considering its essential role in NMD. We subsequently introduce possible variations of this model to accommodate that there are branches of NMD that differ in their requirement for particular NMD factors.

Newly synthesized intron-containing pre-mRNAs, bound at their 5′-caps by the nuclear cap-binding complex (CBC) that consists of a heterodimer of cap-binding protein (CBP)80 and CBP20 (Maquat et al. 2010; Gonatopoulos-Pournatzis and Cowling 2014; Müller-McNicoll and Neugebauer 2014), generally undergo cotranscriptional splicing (Nojima et al. 2018). Splicing removes introns and connects exons to generate mRNAs, with the possibility of producing one or more differentially spliced mRNA isoforms depending on cell type or developmental stage (Singh et al. 2015). As a consequence of splicing, a protein complex called the exon-junction complex (EJC) is deposited onto newly synthesized mRNAs ∼20–24 nt upstream of the resulting exon−exon junctions with an estimated efficiency of 80% (Le Hir et al. 2000, 2016; Bono et al. 2006; Singh et al. 2012; Boehm and Gehring 2016; Woodward et al. 2017). Once matured at their 3′-ends by endonucleolytic cleavage and polyadenylation that, like splicing, can produce alternative mRNA isoforms, the resulting mature mRNAs are exported from the nucleus to cytoplasm carrying the CBC, EJCs, and other RNA-binding proteins (Maquat et al. 2010; Gonatopoulos-Pournatzis and Cowling 2014; Müller-McNicoll and Neugebauer 2014). In the cytoplasm, mRNAs continue to undergo dramatic remodeling of their associated proteins, including replacement of the CBC by the cytoplasmic CBP eukaryotic translation initiation factor (eIF) 4E and loss of the EJCs (Sato and Maquat 2009; Maquat et al. 2010). Of particular relevance to NMD, the CBC not only remains bound to 5′-caps during mRNA export to the cytoplasm (Ishigaki et al. 2001; Lejeune et al. 2002), but it also recruits ribosomes to support the pioneer, or first, round of mRNA translation in the cytoplasm (Ishigaki et al. 2001; Kim et al. 2009; Choe et al. 2012, 2014b).

There are two categories of NMD, and both are tightly linked to the process of protein synthesis, during which termination codons are recognized. NMD that involves an EJC situated downstream from a termination codon, i.e., 3′-untranslated region (3′UTR) EJC-dependent NMD, largely occurs during the translation of newly synthesized, CBC-bound mRNAs undergoing the pioneer round of translation. Depending on the efficiency of translation initiation and the length of the open reading frame, the pioneer round of translation may involve mRNAs that are bound by one or more ribosomes (Chiu et al. 2004; Isken and Maquat 2007; Isken et al. 2008; Kim et al. 2009; Apcher et al. 2011; Sharma et al. 2012). While it is possible that the fraction of 3′UTR EJC-dependent NMD substrates that escapes decay while bound by the CBC may be degraded later, during the subsequent rounds of translation mediated by eIF4E (Durand and Lykke-Andersen 2013; Rufener and Mühlemann 2013), the fraction of the total pool of NMD substrates that is degraded while bound by CBC relative to by eIF4E remains unclear. That noted, single-molecule studies showed that decay occurs rapidly once an mRNA is exported to the cytoplasm, possibly before CBC is replaced by eIF4E (Trcek et al. 2013).

In addition to 3′UTR EJC-dependent NMD, there is NMD that occurs independent of a 3′UTR EJC, often as a consequence of translation termination at the normal termination codon rather than at a PTC (Wang et al. 2002; Bühler et al. 2006; Matsuda et al. 2007; Eberle et al. 2008). Cells define whether or not a termination codon (TC) triggers either type of NMD depending on the ability of UPF1 to be recruited and phosphorylated downstream from the TC (He and Jacobson 2015; Karousis et al. 2016; Kurosaki et al. 2019).

When an elongating ribosome reaches a TC that does not trigger NMD, as typifies most normal TCs, translation termination is relatively efficient. A ternary complex consisting of eukaryotic release factor (eRF)1, eRF3, and GTP is recruited to the ribosomal A site (Dever and Green 2012; Joazeiro 2017; Hellen 2018; Schuller and Green 2018). Once eRF1 binds the TC, GTP hydrolysis by eRF3 induces a conformational change in eRF1 and consequently activates hydrolysis of the polypeptide from the polypeptidyl-tRNA in the ribosomal P site. These processes are promoted by cytoplasmic poly(A)-binding protein 1 (PABPC1) binding to the amino-terminal region of eRF3 (Hoshino et al. 1999; Uchida et al. 2002; Ivanov et al. 2008) independently of GTP hydrolysis by eRF3 and polypeptide release by eRF1 (Ivanov et al. 2016). In addition, release of the polypeptide, eRF3, and GDP is coupled via eRF1 to recruitment of the ATP-binding cassette subfamily E member 1 (ABCE1; also called RNase L inhibitor 1, RLI1), which leads to dissociation of the 60S ribosomal subunit from the 40S ribosomal subunit and facilitates efficient ribosome recycling (Khoshnevis et al. 2010; Pisarev et al. 2010; Dever and Green 2012).

However, when an elongating ribosome reaches a TC that does trigger NMD, translation termination is relatively inefficient because of factors that delay the process. In the case of 3′UTR EJC-dependent NMD, the EJC delays termination. Delay is in part due to the binding of UPF1 and its phosphatidylinositol 3-kinase-related kinase SMG1 to the terminating ribosome along with the eRF1–eRF3 complex, forming the SURF complex (SMG1–UPF1–eRF1–eRF3; Kashima et al. 2006). The binding of UPF1 to eRF3 in SURF appears to out-compete binding of the termination-promoting factor PABPC1 to eRF3 (Singh et al. 2008). Subsequently, the amino-terminal cysteine- and histidine-rich domain of UPF1 interacts with the carboxy-terminal region of EJC-bound UPF2 (Weng et al. 1996; Serin et al. 2001; Kadlec et al. 2006; Chamieh et al. 2008), whose third middle domain of eIF4G (MIF4G) domain interacts with the amino-terminal ribonucleoprotein-type RNA-binding domain of the EJC component UPF3X (Serin et al. 2001; Kadlec et al. 2004; Chamieh et al. 2008). Here again, interactions between NMD factors at the TC and the EJC reduce the efficiency of termination by antagonizing the termination-promoting contributions of PABPC1 (Eberle et al. 2008; Ivanov et al. 2008; Singh et al. 2008). This leads to the formation of an mRNA decay-inducing complex (DECID), which is promoted by the RNA helicase DHX34 (Hug and Cáceres 2014; Melero et al. 2016), and UPF1 activation by SMG1-mediated phosphorylation (Kashima et al. 2006; Kurosaki and Maquat 2013; Kurosaki et al. 2014; Durand et al. 2016). In the case of 3′UTR EJC-independent NMD, UPF1 is preferentially bound to the 3′UTR either because of a 3′UTR structure that impedes 5′-to-3′ UPF1 helicase activity or because the 3′UTR is sufficiently long to generate a higher probability of being occupied by promiscuously bound UPF1 (Zünd et al. 2013; Imamachi et al. 2017). Thus, in both 3′UTR EJC-dependent NMD and 3′UTR EJC-independent NMD, termination-delaying (i.e., NMD-stimulating) factor(s) that result from UPF1 recruitment to, respectively, a 3′UTR EJC or the 3′UTR itself, outcompete termination-promoting (i.e., NMD-antagonizing) factor(s) such as PABPC1. The effect of these termination-delaying factors may be overridden by the binding of a cellular protein, such as polypyrimidine tract binding protein or hnRNP L, immediately downstream from the termination codon (Ge et al. 2016; Kishor et al, 2018). Notably, once UPF1 is phosphorylated, the NMD substrate is further remodeled so that additional translation initiation events are precluded and mRNA degradative activities are recruited, both of which are required for mRNA decay (see below).

An alternative mechanism by which a TC could be recognized was recently proposed based on assays of translation termination in vitro using recombinant proteins (Neu-Yilik et al. 2017). Instead of finding a role for UPF1 in translation termination, it was discovered that translation termination was delayed by the direct interaction of UPF3X with eRF3, and that UPF3X directly binds to UPF1 even in the absence of UPF2, consistent with previous data showing that cellular UPF3X and UPF1 coimmunoprecipitate in the absence of UPF2 (Gong et al. 2009). The in vitro experiments used limiting amounts of eRF1 and eRF3 and an excess of UPF3X with the goal of recapitulating the inefficient translation termination that is observed during cellular NMD but simultaneously complicating interpretations. Nevertheless, these experiments might provide molecular clues to the branch of cellular 3′UTR EJC-dependent NMD that relies on UPF1 and UPF3X but not UPF2 (Gehring et al. 2005; Chan et al. 2007).

## NMD: SELECTIVE SUBSTRATE DEGRADATION

Once UPF1 undergoes phosphorylation, it inhibits further translation initiation events on the NMD substrate and recruits mRNA decay activities. Translation inhibition is mediated by phosphorylated UPF1 binding to the two highest molecular weight subunits of eIF3 that constitute the 43S preinitiation complex poised at the initiating AUG codon: Binding precludes 60S ribosomal subunit joining to the 43S preinitiation complex so as to prevent formation of a translationally active 80S ribosome (Isken et al. 2008). Without translational repression, NMD fails to occur (Isken et al. 2008). Phosphorylated UPF1 additionally recruits mRNA degradative activities both directly, as exemplified by SMG6, and via other NMD factors, including the heterodimer SMG5−SMG7 and/or the protein-rich nuclear receptor coactivator 2 (PNRC2) (Anders et al. 2003; Chiu et al. 2003; Ohnishi et al. 2003; Unterholzner and Izaurralde 2004; Fukuhara et al. 2005; Cho et al. 2009; Eberle et al. 2009; Okada-Katsuhata et al. 2012). Given the propensity for steady-state hypophosphorylated UPF1 to bind any cellular RNA that is physically accessible (Hogg and Goff 2010; Kurosaki and Maquat 2013; Zünd et al. 2013; Kurosaki et al. 2014), binding by phosphorylated UPF1 provides a reliable molecular identifier of cellular NMD substrates (Kurosaki et al. 2014, 2018; Durand et al. 2016; Imamachi et al. 2017).

Mammalian-cell NMD involves both endoribonucleolytic and exoribonucleolytic activities (Chen and Shyu 2003; Lejeune et al. 2003; Couttet and Grange 2004; Huntzinger et al. 2008; Eberle et al. 2009). The endoribonuclease SMG6 cleaves mRNA close to the TC, resulting in 5′-cleavage fragments and 3′-cleavage fragments, the latter of which are still polyadenylated (Eberle et al. 2009). These fragments are subsequently degraded by, respectively, the 3′-to-5′ multisubunit exosome and the 5′-to-3′ exoribonuclease XRN1 (Huntzinger et al. 2008; Eberle et al. 2009). Exoribonucleolytic decay by deadenylation is mediated by SMG5−SMG7: A CCR4–NOT deadenylase complex is recruited via a direct interaction between SMG7 and the POP2 catalytic subunit of the CCR4–NOT deadenylase complex, eliciting deadenylation followed by exosome-mediated 3′-to-5′ degradation (Loh et al. 2013). Decapping followed by 5′-to-3′ degradation is activated by PNRC2 in concert with or independently of the SMG7–POP2 interaction (Cho et al. 2009, 2013a; Loh et al. 2013). PNRC2 binds DCP1A, a component of the decapping complex, to elicit decapping, which is followed by 5′-to-3′ exoribonucleolytic cleavage (Cho et al. 2009; Lai et al. 2012; Loh et al. 2013; Mugridge et al. 2016; Nicholson et al. 2018). Since (i) artificially tethered SMG5 causes rapid degradation of reporter mRNAs in a PNRC2-dependent and SMG7-dependent manner (Nicholson et al. 2018), and (ii) PNRC2 preferentially associates with SMG5 rather than SMG6 or SMG7 (Cho et al. 2013a), it is likely that SMG5 forms two mutually exclusive complexes: SMG5–SMG7 or SMG5–PNRC2. The resulting alternative pathways may contribute to the observed preference of particular NMD substrates for particular decay-inducing factors downstream from UPF1 phosphorylation (Kurosaki et al. 2018). A structural analysis revealed that PNRC2 bridges the interaction between DCP1A and the DCP2 decapping enzyme, synergistically acting with DCP1A to stimulate DCP2 decapping activity (Lai et al. 2012). This, together with a recent kinetic analysis of PNRC2 revealing that a conserved short linear motif in PNRC2 enhances both substrate binding and the catalytic step of decapping (Mugridge et al. 2016), suggests that PNRC2 functions during NMD as a decapping co-activator and an adaptor that links UPF1 to the decapping complex (Cho et al. 2009; Lai et al. 2012; Nicholson et al. 2018).

Recent transcriptome-wide characterizations of NMD decay intermediates have uncovered additional aspects of the degradative process (Kurosaki et al. 2018). For one, NMD decay intermediates can be uridylated at their 3′-ends by the terminal uridylyl transferases, TUT4 and TUT7. This appears to promote subsequent 3′-to-5′ exoribonucleolytic decay by either the DIS3-like 3′-to-5′ exoribonuclease DIS3L2 or the exosome, depending on the length of terminally added uridine nucleotides. Nontemplated 3′-end uridines are also known to trigger decapping followed by XRN1-mediated 5′-to-3′ degradation by recruiting the LSM1-7 complex (Song and Kiledjian 2007; Rissland and Norbury 2009). Data indicate that oligouridylated NMD decay intermediates are bound not only by phosphorylated UPF1 but also by one or more ribosomes (Kurosaki et al. 2018). Moreover, the 3′-end addition of a nontemplated nucleotide other than uridine inhibits decay (Kurosaki et al. 2018).

## NMD: MOLECULAR ACTIVITIES OF UPF1

**FIGURE 2.**
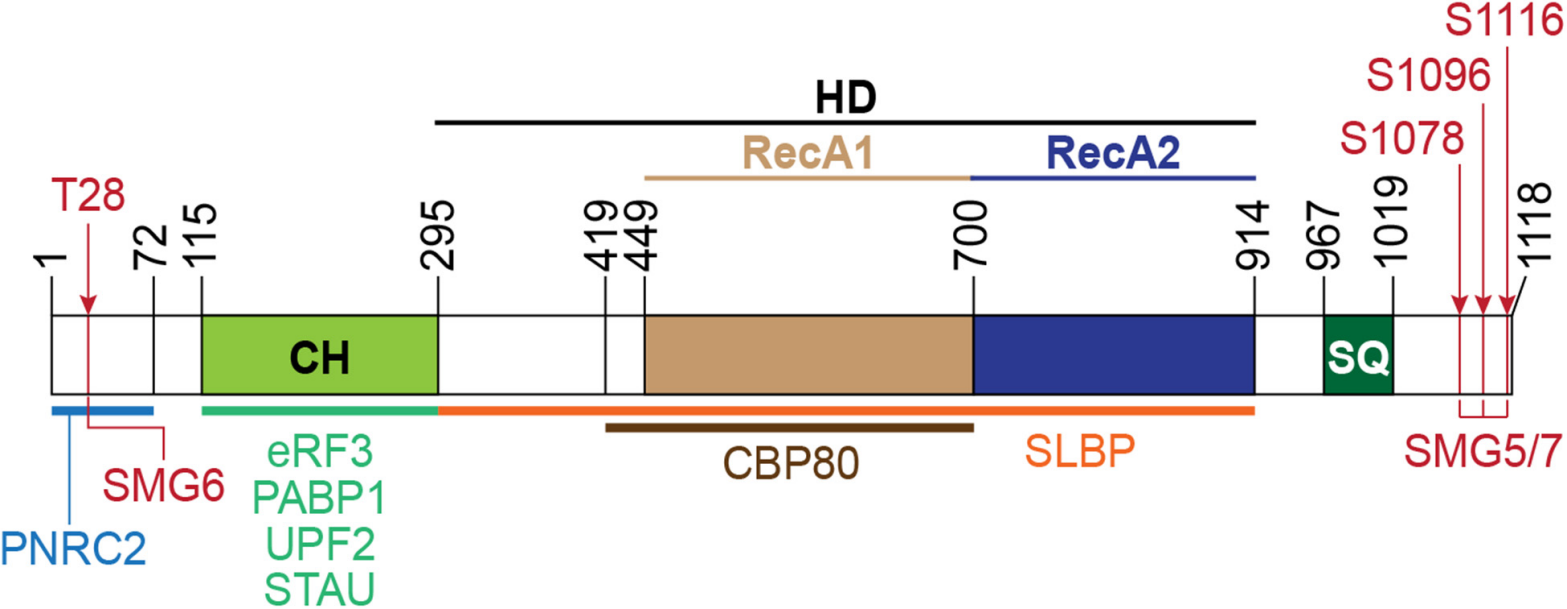
Schematic of UPF1 domains. The binding regions for UPF1-interacting proteins are indicated. Numbers and red arrows indicate amino acid positions and experimentally validated phosphorylation sites, respectively. CH, cysteine- and histidine-rich domain; HD, helicase domain; and SQ, serine- and glutamine-rich domain.

Mammalian UPF1, which belongs to the superfamily 1 of RNA helicases, exhibits RNA-dependent ATPase and 3′-to-5′ helicase activities, both of which are crucial for NMD (Weng et al. 1996; Franks et al. 2010; Kurosaki et al. 2014). UPF1 is composed of three distinct domains: a cysteine- and histidine-rich (CH) domain situated immediately downstream from an amino-terminal unstructured region, a helicase domain toward the middle, and a carboxy-terminal serine- and glutamine-rich (SQ) domain (Fig. 2).

The CH domain folds into the helicase domain to inhibit the ATPase and RNA helicase activities (Kadlec et al. 2006; Chamieh et al. 2008; Fiorini et al. 2013). During NMD, an interaction between UPF1 and UPF2 displaces the CH domain, inducing a large conformational change in UPF1 that derepresses the inhibitory effect of the CH domain (Chamieh et al. 2008; Chakrabarti et al. 2011).

The helicase domain, which consists of two RecA-like domains, associates with ATP via the cleft situated between the two RecA-like domains (Chakrabarti et al. 2011; Fiorini et al. 2013). Recent data obtained using magnetic tweezers revealed that the helicase domain unwinds long double-stranded nucleic acids and translocates along single-stranded nucleic acids with high processivity (Fiorini et al. 2015). The tight grip of UPF1, which allows its high processivity, was recently proven to be essential for NMD (Kanaan et al. 2018).

Many [S/T]Q motifs in the amino-terminal unstructured region and the carboxy-terminal SQ domain of UPF1 become phosphorylated by the SMG1 kinase after TC recognition that triggers either 3′UTR EJC-dependent or 3′UTR EJC-independent NMD. As noted above, it is phosphorylated UPF1 that represses additional translation initiation events on the NMD target and recruits the downstream NMD factors (SMG5–SMG7 and/or PNRC2) for the substrate decay steps of NMD. Down-regulation of SMG5, SMG6, SMG7, or PNRC2 causes phosphorylated UPF1 to accumulate, suggesting that inhibiting decay increases the amount of phosphorylated UPF1 (Durand et al. 2016). Extensive mutational analyses revealed that progressive phosphorylation of the various [S/T]Q motifs increases the ability of UPF1 to activate mRNA decay (Durand et al. 2016). While phosphorylation of any particular [S/T]Q motif does not seem to be crucial, a subset of motifs contributes to UPF1 function more significantly than do others (Yamashita et al. 2001; Ohnishi et al. 2003; Unterholzner and Izaurralde 2004; Fukuhara et al. 2005; Kashima et al. 2006). It is likely that phosphorylation at different residues results in a different binding preference for different downstream decay-inducing factors. For instance, UPF1 phosphorylation at residue T28 in the amino terminus or S1096 in the carboxyl terminus preferentially recruits SMG6 or SMG5–SMG7, respectively. Such phosphorylation site-dependent recruitments are known to be required for efficient NMD (Okada-Katsuhata et al. 2012). It remains to be determined if UPF1 phosphorylation at different residues occurs in a sequential or otherwise regulated manner. It is also unknown if the degree to which each of the possible downstream decay-inducing factors functions in NMD depends on what UPF1 residues have undergone phosphorylation. However, as additional evidence that UPF1 phosphorylation at different [S/T]Q motifs results in different outcomes, while UPF1 undergoes phosphorylation by both ATM and SMG1 kinases in cells exposed to ionizing radiation, down-regulating SMG1, unlike down-regulating ATM, inhibits NMD (Brumbaugh et al. 2004).

## UPF1 IN STAUFEN-MEDIATED mRNA DECAY (SMD)

**FIGURE 3.**
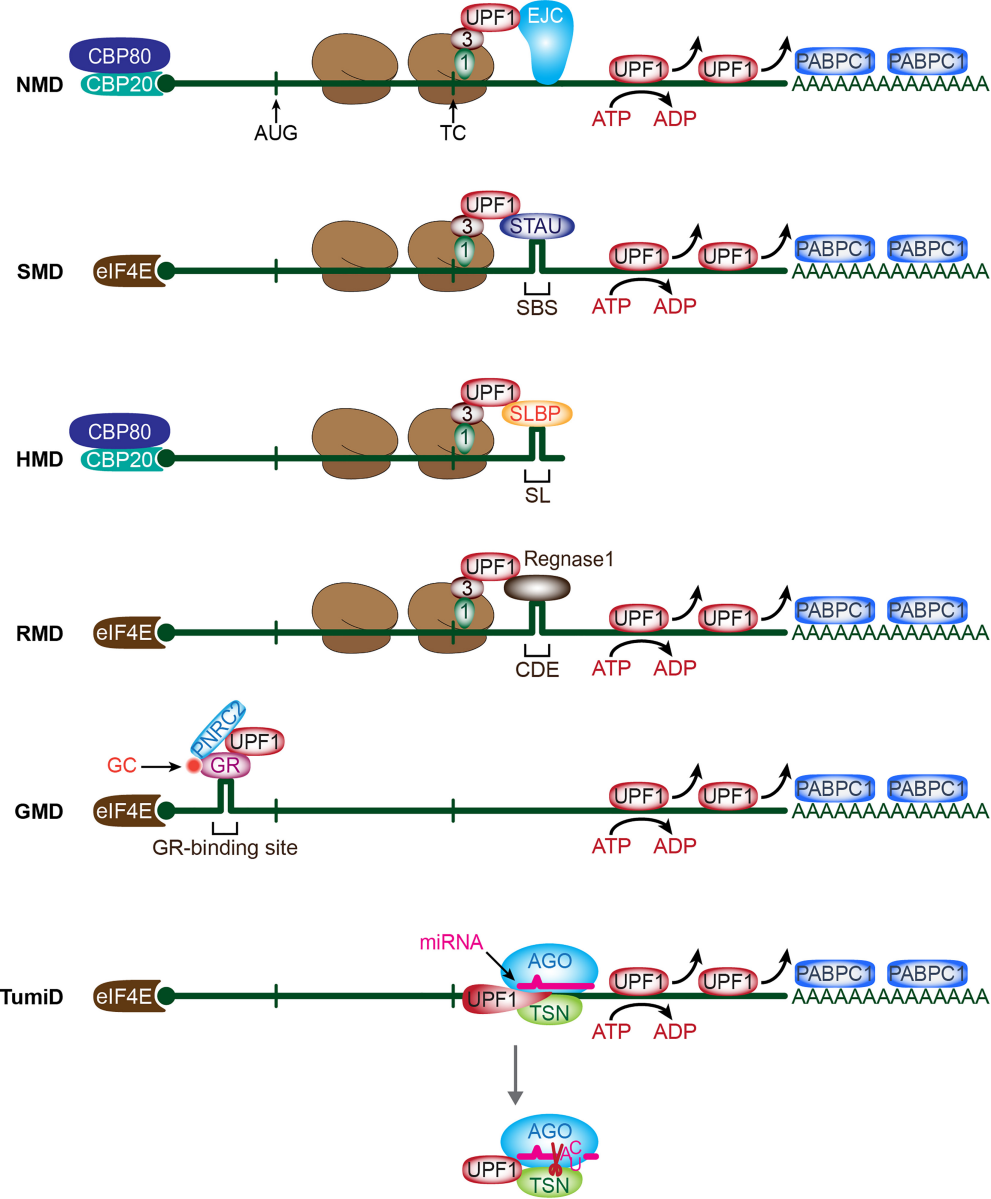
Various UPF1-dependent RNA decay pathways. Shown are six categories of RNA decay pathways that differ by how UPF1 engages with the substrate. UPF1 is engaged in NMD via either a 3′UTR EJC or another 3′UTR feature that attracts UPF1 and delays translation termination. UPF1 is engaged in SMD via 3′UTR-bound STAU1 or STAU2, and in HMD via a 3′UTR SLBP. GMD engages UPF1 via a GC-bound GR and RMD via regnase 1. Finally, TumiD engages UPF1 as a transiently or weakly associated constituent of the RNA-induced silencing complex, which consists of an AGO protein. Each of these pathways require the helicase activity of UPF1 and, of the mRNA decay pathways, all but GMD require the substrate be translated. The primary cap-binding protein(s) for each pathway are shown. SBS, STAU-binding site; SL, histone stem–loop; CDE, constitute decay element.

In NMD, UPF1 enrichment at a 3′UTR, whether the 3′UTR harbors an EJC or not, results in UPF1 joining to an upstream terminating ribosome. It is also possible that UPF1 recruitment to a terminating ribosome is mediated by an RNA-binding protein that simultaneously binds a 3′UTR and UPF1. This mode of recruitment typifies mRNA decay that is mediated by the double-stranded RNA-binding protein staufen (STAU)—in mammals, either STAU1 or STAU2—so as to trigger STAU-mediated mRNA decay (SMD) (Table 1; Fig. 3; Kim et al. 2005). Originally, STAU was identified as a cellular factor that functions to determine the spatiotemporal localization of maternal mRNAs in *Drosophila* oocytes and eggs (Broadus et al. 1998; Roegiers and Jan 2000; Park and Maquat 2013; Heraud-Farlow and Kiebler 2014). In mammals, STAUs are also involved in mRNA localization in, e.g., neurons and oocytes (Kiebler et al. 1999; Tang et al. 2001; Miki et al. 2005; Maquat and Gong 2009). Additionally, when STAU1 (Kim et al. 2005) or STAU2 (Park et al. 2013) binds to an mRNA 3′UTR, it can trigger translation-dependent and UPF1-dependent SMD. SMD, like NMD, targets diverse transcripts and participates in diverse cellular and physiological processes including myogenesis, adipogenesis, cell motility, and autophagy (Kim et al. 2005, 2007; Gong et al. 2009; Maquat and Gong 2009; Gong and Maquat 2011; Cho et al. 2012; Park and Maquat 2013; Paul et al. 2018).

**TABLE 1.**
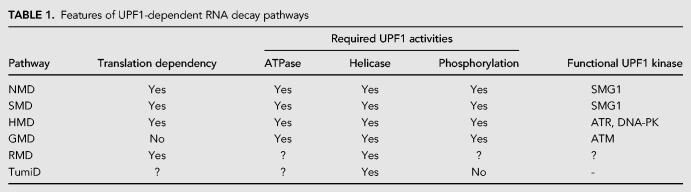
Features of UPF1-dependent RNA decay pathways

In SMD, substrate selection is determined by STAU binding to a 3′UTR double-stranded structure, a so-called STAU-binding site (SBS), in target mRNAs (Kim et al. 2005, 2007). SBSs can be generated with an SMD target by either intramolecular base-pairing within the 3′UTR or intermolecular base-pairing between a short interspersed nuclear element (SINE) within the 3′UTR and a partially complementary SINE within one or more computationally defined long noncoding RNAs, termed 1/2-sbsRNAs, and/or one or more mRNAs (Kim et al. 2005, 2007; Gong and Maquat 2011; Gong et al. 2013; Lucas et al. 2018). If base-pairing occurs between two mRNAs via their 3′UTR SINEs, e.g., Alu elements in humans, then both mRNAs are targeted by SMD; however, if base-pairing occurs between the 3′UTR SINE of an mRNA and a 1/2-sbsRNA that fails to undergo translation, only the mRNA is targeted for SMD (Bono et al. 2006; Gong and Maquat 2011; Gong et al. 2013).

SMD involves SBS-bound STAU directly recruiting UPF1 downstream from a TC, much as the EJC recruits UPF1 during NMD. However, since SMD does not involve an EJC, SMD is not restricted to newly synthesized mRNAs. While uncertain, it is likely that the mechanisms of SMD and NMD converge upon UPF1 activation by SMG1-mediated phosphorylation (Cho et al. 2013b). Therefore, it can be postulated that when an elongating ribosome reaches a TC, recruitment of UPF1 downstream from the TC—in the case of SMD, to the 3′UTR via its direct interaction with STAU—links the termination event to mRNA decay. As is the case for NMD, where UPF2 displaces the CH domain of UPF1 and induces a conformational change in UPF1, STAU1 interacts with the CH domain of UPF1 (Gong et al. 2009) and does likewise, activating UPF1 ATP hydrolysis and helicase activity (Park et al. 2013) and triggering UPF1 phosphorylation by SMG1 (Cho et al. 2013b). After that, phosphorylated UPF1 recruits PNRC2 that, as it does during NMD, elicits decapping followed by 5′-to-3′ exoribonucleolytic cleavage of SMD-targeted mRNAs (Cho et al. 2009, 2012). It is currently unknown whether other decay-inducing factors, e.g., SMG5, SMG6, and SMG7 that engage in NMD are involved in SMD or whether the same or different [S/T]Q motifs in UPF1 are phosphorylated during SMD and NMD.

## UPF1 IN REPLICATION-DEPENDENT HISTONE mRNA DECAY (HMD)

Mammalian cell proliferation requires a proper balance between the amounts of DNA and histone proteins. Balance is in part achieved by controlling the stabilities and translational efficiencies of replication-dependent histone mRNAs, which typically lack the poly(A) tail found at the 3′-ends of most eukaryotic mRNAs (Marzluff et al. 2008; Marzluff and Koreski 2017). Instead, of a poly(A) tail, these mRNAs harbor an evolutionarily conserved 3′UTR stem–loop structure (Jaeger et al. 2005; Hoefig and Heissmeyer 2014) that is crucial for their rapid degradation at the end of the S phase of the cell cycle, i.e., after DNA has been duplicated, or under stressful conditions where DNA replication is inhibited (Sittman et al. 1983; Pandey and Marzluff 1987; Harris et al. 1991). Remarkably, the overall shape of this stem–loop structure is unique and specifically recognized by stem–loop-binding protein (SLBP; Tan et al. 2013). SLBP performs pivotal functions at multiple steps in the biogenesis of replication-dependent histone mRNAs, including pre-mRNA processing, mRNA export, translation, and degradation (Marzluff et al. 2008; Hoefig and Heissmeyer 2014; Marzluff and Koreski 2017).

SLBP-mediated replication-dependent histone mRNAs decay (HMD) is mechanistically reminiscent of NMD and SMD (Table 1; Fig. 3) in that HMD depends on translation and UPF1 recruitment downstream from the TC. Under normal conditions, CBC-dependent translation initiation factor (CTIF) preferentially associates with SLBP (Choe et al. 2013, 2014a), thus allowing for the increased stability and efficient translation of replication-dependent histone mRNAs (Choe et al. 2014a). Under stressful conditions that result in a DNA replication block, the phosphatidylinositol 3-kinase-related kinases ATR and DNA-PK are activated and phosphorylate UPF1 (Kaygun and Marzluff 2005; Choe et al. 2014a). Although it is unknown whether these kinases target free UPF1, SLBP-bound UPF1 or UPF1 in the SURF complex (Kaygun and Marzluff 2005), UPF1 phosphorylation, which is critical for HMD (Kaygun and Marzluff 2005), enhances UPF1 binding to SLBP and disrupts the interaction between SLBP and CTIF, thereby leading to the translational suppression of replication-dependent histone mRNAs (Choe et al. 2014a). Since HMD does not involve either UPF2 or STAU, possibly dissociation of the inhibitory interaction between the CH domain and helicase domain of UPF1 during HMD may be mediated by the amino-terminal half of SLBP, which interacts directly with the helicase domain of UPF1 and promotes UPF1 ATPase and helicase activities (Choe et al. 2014a). As another possibility, phosphorylation of the UPF1 [S/T]Q motifs by ATR or DNA-PK may be sufficient to trigger the large conformational change of UPF1 that is required to promote the helicase activity of UPF1, either by displacing the CH domain or by generating a platform favorable for SLBP binding.

The complex of SLBP and phosphorylated UPF1 preferentially recruits SMG5 and PNRC2, thereby triggering replication-dependent histone mRNA decapping followed by 5′-to-3′ exoribonucleolytic decay (Choe et al. 2014a). In contrast to NMD, down-regulating either SMG6 or SMG7 does not significantly alter the efficiency of HMD (Choe et al. 2014a), suggesting that SLBP may influence which degradative activities are recruited. As an additional difference between HMD and NMD, decapping followed by 5′-to-3′ exoribonucleolytic degradation during HMD is induced by an alternative pathway either simultaneously with or independently of PNRC2. Moreover, oligouridylation during HMD primarily by TUT7 and/or possibly TUT4 promotes 3′-to-5′ degradation, starting downstream from the stem–loop and continuing into the translated region, where ribosomes remain bound (Mullen and Marzluff 2008; Schmidt et al. 2011; Su et al. 2013; Lackey et al. 2016). As proposed for NMD, oligouridylated terminal sequences in histone mRNAs also provide a platform for loading the LSM1-7 complex, which activates decapping followed by XRN1-mediated 5′-to-3′ degradation (Song and Kiledjian 2007; Rissland and Norbury 2009). It is quite likely that, like NMD and HMD, many if not all decay pathways that proceed from an mRNA 3′-end involve oligouridylation to overcome higher-order RNA structural barriers to the 3′-to-5′ exosome.

## UPF1 IN GLUCOCORTICOID RECEPTOR-MEDIATED mRNA DECAY (GMD)

Glucocorticoid (GC) receptor (GR) is a DNA-binding transcription factor that belongs to the nuclear receptor superfamily and regulates various biological and physiological processes (Oakley and Cidlowski 2011; Santos et al. 2011; Vandevyver et al. 2012). Although it has long been appreciated that GR targets DNA, several studies have revealed that GR also functions as an RNA-binding protein to elicit rapid degradation of target substrates (Dhawan et al. 2007, 2012; Kino et al. 2010; Ishmael et al. 2011; Cho et al. 2015; Park et al. 2015, 2016) in a process called GR-mediated mRNA decay (GMD) (Table 1; Fig. 3).

Recent biochemical and transcriptome-wide analyses point to a two-step model for GMD: mRNA recognition by GR in the absence of a GC and, subsequently, rapid mRNA decay in the presence of a GC (Cho et al. 2015; Park et al. 2016). Unlike GR binding to DNAs, which occurs in response to a GC ligand, GR binding to RNAs occurs independent of a GC ligand. When cells are treated with a GC, the GR preexisting on target mRNAs associates with the GC, after which the GC–GR complex recruits PNRC2 (Cho et al. 2015; Park et al. 2016). The resulting complex is functionally inactive but relatively stable. It contains PNRC2 that appears to provide a binding platform for UPF1 and DCP1A because down-regulating PNRC2 abrogates the coimmunoprecipitation of UPF1 and DCP1A with GR (Cho et al. 2015; Park et al. 2016). Subsequent recruitment of Y-box-binding protein 1 (YBX1) and the endoribonuclease heat-responsive protein (HRSP)12 (also known as reactive intermediate imine deaminase A homolog, or RIDA) activates the complex so as to elicit efficient GMD via PNRC2-mediated decapping followed by 5′-to-3′ exoribonucleolytic cleavage (Park et al. 2016). As with other mRNA decay pathways, GMD may also involve 3′-to-5′ exoribonucleolytic cleavage. Additionally, the known endoribonucleolytic activity of HRSP12 (Morishita et al. 1999; Mistiniene et al. 2003, 2005) may function in GMD.

Although GMD, NMD, SMD, and HMD involve UPF1 and PNRC2, GMD stands apart from the other pathways in several ways. First, unlike NMD, SMD, and HMD, GMD occurs independent of mRNA substrate translation: Blocking translation initiation using a strong hairpin structure in the 5′UTR does not alter GMD efficiency (Cho et al. 2015). Second, although NMD, SMD, and HMD involve communication between a terminating ribosome on a TC and cellular factors loaded onto the 3′UTR of target mRNAs (the EJC or another 3′UTR feature that results in UPF1 loading, STAU or SLBP, respectively), GMD does not require the GR-binding site to be in the 3′UTR (Cho et al. 2015). Indeed, the first identified cellular GMD substrate, *CCL2* mRNA, contains a GR-binding site in the 5′UTR (Cho et al. 2015). Third, GMD is a ligand-inducible mRNA decay pathway. GC treatment is sufficient for eliciting efficient GMD, as long as GMD substrates contain GR-occupied GR-binding sites (Cho et al. 2015; Park et al. 2015, 2016).

GMD manifests additional interesting features. GMD requires PNRC2, which interacts with the unstructured amino terminus of UPF1 (i.e., amino acids 1–72), the ATPase and helicase activities of UPF1 (Park et al. 2016), and UPF1 phosphorylation (Park et al. 2016). Whereas NMD preferentially utilizes SMG1-mediated UPF1 phosphorylation, GMD is not dependent on SMG1 (Cho et al. 2015) but instead preferentially uses ATM-mediated UPF1 phosphorylation (Park et al. 2016). As noted above, this suggests that SMG1 and ATM may phosphorylate UPF1 at different [S/T]Q motifs in the amino-terminal region and in the carboxy-terminal SQ domain so as to generate binding platforms for different decay-inducing factors.

## REGNASE 1-MEDIATED mRNA DECAY (RMD)

Coordinated transcriptional and post-transcriptional regulation of gene expression in response to inflammation is crucial for immune homeostasis (Chovatiya and Medzhitov 2014). Many inflammatory mRNAs are very unstable because they contain a conserved 3′UTR *cis*-acting element, such as an AU-rich element (ARE) or a constitutive decay element (CDE), the latter of which forms a hairpin structure harboring a pyrimidine–purine–pyrimidine loop (Stoecklin et al. 2003; Glasmacher et al. 2010; Leppek et al. 2013). ARE-containing mRNAs are rapidly degraded in innate-immune cells in general, although they are stabilized upon activation of a set of pattern-recognition receptors, such as Toll-like receptors (Takeuchi and Akira 2010). The stability of ARE-containing mRNAs is determined by the ARE-mediated binding of *trans*-acting cellular factors that either stabilize or destabilize the mRNA. These factors include Hu antigen R (HuR; also known as embryonic lethal abnormal vision-like 1, ELAVL1), tristetraprolin (TTP), butyrate response factors 1 (BRF1) and BFR2, KH-type splicing regulatory protein (KSRP), and AU-rich element RNA-binding protein 1 (AUF1) (Gratacós and Brewer 2010; von Roretz et al. 2011; Schoenberg and Maquat 2012; Brooks and Blackshear 2013; Grammatikakis et al. 2017; Khabar 2017; Mayr 2017).

mRNAs harboring a CDE are selectively recognized and rapidly degraded by the ring finger- and CCCH-type domain-containing protein known as roquin (Leppek et al. 2013). Recent studies revealed that 3′UTR CDEs are also recognized by the zinc finger CCCH-type containing 12a protein known as regnase 1 (also known as ZC3H12A, and originally identified as monocyte chemotactic protein-induced protein 1; Mino et al. 2015; Takeuchi 2018). Regnase 1 contains a PilT amino-terminal (PIN)-like RNase domain (also called a NYN domain) that manifests endoribonuclease activity (Matsushita et al. 2009).

Although regnase 1 and roquin commonly recognize a 3′UTR, the molecular mechanisms underlying mRNA destabilization are quite different (Leppek et al. 2013; Mino et al. 2015). CDE-bound roquin initiates rapid mRNA degradation by recruiting a CCR4–NOT deadenylase complex, while regnase 1-mediated mRNA decay (RMD) proceeds in a UPF1- and a translation-dependent manner (Table 1; Fig. 3). Regnase 1 and roquin are in charge of tightly regulating inflammatory mRNA decay in acute and late phases of inflammation, respectively.

In terms of the mechanism, RMD, like NMD and SMD, depends on translation termination. In RMD, 3′UTR-bound regnase 1 appears to be functionally equivalently to those 3′UTR features that trigger NMD and SMD (see above). Taking cues from NMD, when an elongating ribosome reaches a TC during RMD, UPF1 may be recruited to the terminating ribosome either via the SURF complex or through promiscuous preassociation with the 3′UTR of the target mRNA. UPF1 in the terminating ribosome may bridge the translation termination complex and downstream CDE-bound regnase 1 (Leppek et al. 2013; Mino et al. 2015). This could subsequently trigger regnase 1 endoribonuclease activity and can activate UPF1 helicase activity, which is critical for efficient RMD (Leppek et al. 2013). Whether RMD requires UPF1 phosphorylation and other decay-inducing factors recruited by UPF1 has yet to be tested.

## UPF1 IN TSN-MEDIATED microRNA DECAY (TumiD)

MicroRNAs (miRNAs), which are short noncoding RNAs of ∼22 nt, mediate gene silencing by guiding a miRNA-induced silencing complex (miRISC) to miRNA-binding sites in the 3′UTR of target mRNAs (Duchaine and Fabian 2018; Gebert and MacRae 2019). A miRNA-loaded miRISC then triggers translation repression and possibly also degradation of target mRNAs. Although tremendous discoveries have expanded our understanding of miRNA biogenesis and function, research into miRNA decay is in its youth.

miRNA stability largely depends on the miRNA sequence itself and the extent of base-pairing to its various target mRNAs (Duchaine and Fabian 2018; Gebert and MacRae 2019). miRNA stability is also affected by environmental cues and differs among different cell types. A recent study showed that TSN, an endonuclease responsible for degrading A-to-I edited double-stranded RNAs (Nishikura 2016), also degrades a subset of miRNAs that harbor CA or UA dinucleotides in a process termed TSN-mediated miRNA decay or TumiD (Table 1; Fig. 3; Elbarbary et al. 2017b). While UPF1 is not required in vitro to degrade TumiD targets that either are or are not associated with argonaute 2 (AGO2), which is a core component of miRISCs (Elbarbary et al. 2017b), UPF1 promotes TumiD in cells by dissociating miRNAs from their mRNA targets, rendering the miRNAs more susceptible to decay (Elbarbary et al. 2017a). TumiD requires UPF1 helicase activity but is not influenced by UPF1 phosphorylation (Elbarbary et al. 2017a). How UPF1 helicase activity is derepressed during TumiD is currently unknown.

## CONCLUSIONS

It has long been recognized that UPF1 plays a central role in NMD. Moreover, as outlined in this review, accumulating evidence indicates that UPF1 contributes to various RNA decay pathways that include SMD, HMD, GMD, RMD, and TumiD. It can be expected that changes in the cellular concentration of UPF1, which often occur during cellular development, differentiation and stress, will affect the various UPF1-dependent RNA decay pathways to different extents. It also should be noted that different cell types can manifest differences in the relative efficiencies of each UPF1-dependent pathway. For example, relative to HeLa cells, HEK293T cells manifest only a low degree of SMD, explaining how immunoprecipitation of HEK293T-cell transcripts using antibody to phosphorylated UPF1 readily identifies both 3′UTR EJC-dependent and 3′UTR EJC-independent NMD targets but not SMD targets (Kurosaki et al. 2014). The finding that UPF1-dependent RNA decay pathways can influence one another also complicates their evaluation. For example, competition between STAU1 and UPF2 for binding to UPF1 results in competition between the SMD and NMD pathways (Gong et al. 2009). To complicate matters even further, UPF1 may be involved in previously uncharacterized RNA decay pathways. For instance, PNRC2 binding to nuclear receptors is not limited to the GR (Zhou et al. 2000, 2006; Zhou and Chen 2001), and several nuclear receptors can bind RNA (Lalli et al. 2000; Xu and Koenig 2004; Colley et al. 2008; Colley and Leedman 2011; Hentze et al. 2018). It follows that nuclear receptors in addition to GR may bind to specific RNAs, recruit PNRC2, and consequently elicit UPF1-dependent RNA decay. Moreover, there are undoubtedly other RNA-binding proteins that in the future will be discovered to recruit UPF1 to transcripts, some of which could trigger mRNA decay.

We conclude that UPF1 will be front and center to additional RNA decay pathways, providing cells with novel mechanisms to shape the cellular transcriptome in response to diverse biological or physiological environments.
